# Zebrafish Xenograft Model of Human Lung Cancer for Evaluating Osimertinib Resistance

**DOI:** 10.1155/2019/3129748

**Published:** 2019-06-27

**Authors:** Xin-ying Li, Li-tang Huang, Jia-qi Wu, Ming-fang He, Su-hua Zhu, Ping Zhan, Tang-feng Lv, Yong Song

**Affiliations:** ^1^Department of Respiratory Medicine, Jinling Hospital, Nanjing University School of Medicine, Nanjing, China; ^2^Nanjing University Institute of Respiratory Medicine, Nanjing, China; ^3^Institute of Translational Medicine, College of Biotechnology and Pharmaceutical Engineering, Nanjing Tech University, Nanjing 211816, China

## Abstract

About half of NSCLC patients with EGFR mutation had secondary mutation T790M after treatment with a first-generation tyrosine kinase inhibitor (TKI), Gefitinib. The third-generation of EGFR-TKI Osimertinib is suitable for patients with EGFR mutation and T790M mutation. However, drug screening for NSCLC patients after the emergence of acquired resistance has become a difficult problem for clinicians. In this study, we established drug-resistant cell lines of Gefitinib and Osimertinib to evaluate cell proliferation in vitro. And we investigated the inhibitory effect of different drug concentration gradients on cancer cells. Zebrafish with high homology to human genes were selected as xenotransplantation models to compare the effects of different concentrations of Osimertinib on the proliferation and angiogenesis of zebrafish tumors after transplantation of different lung cancer cell lines. It was confirmed that Osimertinib could inhibit the proliferation of tumor cells with EGFR mutation and T790M resistance mutation in zebrafish, which was consistent with the clinical research conclusion.

## 1. Introduction

According to a report released by the International Agency for Research on Cancer in 2018 [1], lung cancer remains the most common malignant tumor worldwide, with an incidence of 11.6% and a fatality rate of 18.4% among all cancers. Non-small cell lung cancer (NSCLC) accounts for more than 80% of lung cancer, and most patients with NSCLC are advanced when diagnosed. With the development of molecular typing of lung cancer, about two-thirds of patients with non-small cell lung cancer, especially those with therapeutically targeted mutations, have more treatment options and improved survival and prognosis compared with traditional chemotherapy [2]. Gene mutation or fusion of activating epidermal growth factor receptor (EGFR), ERBB1, anaplastic lymphoma kinase (ALK), ROS1 protooncogene receptor tyrosine kinase (ROS1), and serine/threonine protein kinase b-Raf (BRAF) is the most common target in the treatment of NSCLC kinase inhibitors [3], and more and more new driving mutations have been found.

NSCLC patients with EGFR mutation accounted for 10-50%, including exon 19 deletion, exon 21 L858R insertion mutation, and exon 20 mutation [4]. Gefitinib and Erlotinib, as first-generation tyrosine kinase inhibitors (TKIs) targeting EGFR mutations, have been widely used [5, 6], and patients with EGFR mutations are also sensitive to the second-generation inhibitor Afatinib. However, more than 50% of patients with disease progression after the use of the first- and second-generation inhibitors had secondary mutation T790M of EGFR [6]. The third-generation TKIs targeting T790M mutation include Osimertinib (also known as AZD9291) [7], Rociletinib (also known as CO-1686), and WZ4002.

Osimertinib, the third generation of TKIs, which is selective to EGFR tyrosine kinase inhibitor sensitization mutation and T790M resistance mutation, has been shown to be effective in patients with advanced NSCLC [8]. Therefore it has been approved by the FDA for NSCLC patients with EGFR T790M positive mutation [9]. However, a lot of patients still have disease progression after oral administration of Osimertinib [10]. It has been proven that the mutation of EGFR C797S [11] is one of the mechanisms of Osimertinib resistance, but MET amplification, HER2 amplification, activation of RAS signaling pathway, and others are involved in the generation of drug resistance [12].

Patient-derived xenotransplantation (PDX) is a valuable tool in oncology. We can obtain faithful biologically models for different types of cancer and potential platforms for the development of precise oncology methods through xenografts [13]. Previous studies observed the biomarkers related to drug efficacy through PDX model of resected specimens from lung cancer patients and compared the histology, molecular spectrum, and therapeutic response of the original patients. It was found that the response of xenografts to TKI was similar to that of clinical patients [14]. Therefore, xenotransplantation model can be used as a powerful tool to study drug resistance in targeted therapy of NSCLC [15]. However, both bronchoscopic biopsy and CT-guided pulmonary biopsy are invasive examinations, and it is difficult for EGFR-TKI drug-resistant patients with the poor basic condition to tolerate two or three biopsies. It is necessary to cultivate drug-resistant cell lines as donors for xenotransplantation.

Zebrafish has more than 85% homology with human genes [16]. It is a classical model for studying tumors, angiogenesis, drug toxicity evaluation [17], and so on. In addition, zebrafish is transparent to observe at an early stage easily. It has a small volume and grows faster. Compared with animal models such as mice, zebrafish has the advantage of shorter experimental period [18]. Since 2015, some researchers have used zebrafish xenotransplantation model (zPDX) to screen drug sensitivity for acute T-lymphocytic leukemia [19] and multiple myeloma [20]. In the study of solid tumors, Ferreira et al. confirmed that the results of zPDX susceptibility screening for colorectal cancer had an 80% clinical correlation [21].

In this study, we will establish Osimertinib-resistant cell lines and select zebrafish as xenotransplantation model animals to compare the effects of different concentrations of Osimertinib on zebrafish after transplanting different cell lines, in order to evaluate the antitumor effect of Osimertinib.

## 2. Materials and Methods

### 2.1. Chemicals and Reagents

Osimertinib (AZD9291) and Gefitinib were purchased from Selleck Chemicals (Houston, TX, USA). MTT, dimethyl sulfoxide (DMSO), trypan blue solution, and collagenase were obtained from Sigma (St. Louis, MO, USA). Osimertinib and Gefitinib were initially dissolved in dimethyl sulfoxide (DMSO) to stock solutions and further diluted to the desired concentration. Dulbecco's modification of Eagle medium (DMEM), RPMI 1640 medium, fetal bovine serum (FBS), penicillin, and streptomycin were purchased from Gibco Life Technologies (Grand Island, NY, USA).

### 2.2. Cell Lines and Cell Culture

Two lung adenocarcinoma cell lines (PC9 and H1975) were provided by the Institute of Biochemistry and Cell Biology of the Chinese Academy of Sciences (Shanghai, China). PC9 cells were cultured in DMEM medium with 10% fetal bovine serum (FBS) and antibiotics (100 units/ml penicillin and 100 *μ*g/ml streptomycin). H1975 cells were cultured in RPMI 1640 medium supplemented with 10% FBS and antibiotics. All cells were cultured in a 5% carbon dioxide incubator at 37°C.

### 2.3. Establishment of Drug-Resistant Cell Lines

Establishment of Gefitinib-resistant strain PC9-GR was initiated by exposing PC9 cells to Gefitinib at 0.05uM for about 72 hours. Then the cells were transferred to the culture medium without drugs, and the exponential growth cells were screened as drug-resistant cell lines. Following the above steps, the drug concentration was increased from 0.05 *μ*M to 5*μ*M. About six months later, PC9 cells became Gefitinib-resistant strain PC9-GR. The H1975 cell line was cultured in the same way and continuously exposed to Osimertinib to obtain Osimertinib-resistant strain H1975-OR. Subsequently, PC9-GR cells and H1975-OR cells were cultured under the same conditions as PC9 and H1975 cells, respectively.

### 2.4. Cell Cytotoxicity Assay

MTT assay is a classic experiment to observe cell proliferation and cell viability. First, 0.25% trypsin added with EDTA was used to digest the cells in the logarithmic phase, and the concentration of tumor cells was adjusted to 1 × 10^5^/ml. The cell suspension was inoculated into 96-well plate, and 100 UL was added into each hole. 96-well plates were incubated in a 5% carbon dioxide incubator at 37°C for 24 hours. Then, Gefitinib or Osimertinib were dissolved in complete medium and diluted in multiple concentration gradients. 100 ul was added in each hole of the experimental group successively, and incubation continued for 24 h. 96-well plates were removed and 20 ul MTT (5mg/ml) was added to each hole. Incubation was continued for 4 hours, and the supernatant was centrifuged and discarded. 150 ul dimethyl sulfoxide (DMSO) was added to each well and oscillated for 5-10 minutes on a Micro-Oscillator until the crystals were fully dissolved. OD value of each hole was detected by microplate microscopy at a wavelength of 490 mm.

### 2.5. Experimental Animal: Zebrafish Husbandry

All wild zebrafish come from the Animal Model Institute of Nanjing University. The feeding conditions were strictly controlled by temperature 28.5°C, pH (7.4), and salinity. All fish are maintained during the 14-hour bright/10-hour extinction period. The male and female zebrafish were mixed in a latticed container according to the ratio of 2:2 to prevent the eggs from being preyed upon after the embryos were obtained. Abnormal embryos were removed. All zebrafish studies were approved by the Animal Protection and Use Commission (IACUC) of Nanjing University of Technology. The age of embryo was expressed as hours (hpf) and days (dpt) after fertilization.

### 2.6. Cell Injection and Xenograft Zebrafish Models

The fluorescence-labeled medium was prepared by using fluorescent tracer CM-DiI (Invitrogen, Life Technologies, Carlsbad, CA, USA) and added to cell suspension to label PC9, PC9-GR, H1975, and H1975-OR cells. After PBS washing twice, some cells were taken out and counted with trypan blue staining to ensure that the cell survival rate was above 90%. The labeled cells were centrifuged again and suspended in fresh medium to adjust the cell concentration to 2 × 10^7^ cells/ml. Transgenic zebrafish embryos Tg(fli-1: EGFP) of 24 hpf were dehydrogenated by pronase (Sigma-Aldrich, St. Louris, MO, USA). Before injection, 48 HPF embryos treated with deovulated membrane were removed and sucked onto the agar plate after 3-5 minutes of anesthesia. Under the microscope, about 200-300 cells were injected into the yolk sac of each zebrafish embryo after the cells were absorbed by the microinjector. After incubation for 1 hour, the cells were observed under a microscope again to ensure that they were injected into the yolk sac rather than into the circulatory system.

### 2.7. Drug Delivery Test by Microinjection

Osimertinib (AZD9291) was diluted to different concentrations in embryo culture medium for yolk sac microinjection. Before administration, the control group was randomly taken as n=10 fish in EP tube, the egg fluid was sucked up, the collagenase digestion was added, and the embryos were digested overnight at 37°C. Three replicates were set up in each group. One day after injection, the inhibiting effect on the proliferation of zebrafish xenotransplantation model was observed and the dose-effect relationship was counted. The drug group and the control group were placed in a 32°C incubator to avoid light. Fresh oocyte solution with different drug concentration was replaced every 24 hours for 3 consecutive days. Three days after administration (3dpt), embryos were digested according to the above method. Three replicates were set up in each administration group and control group.

### 2.8. Fluorescence Microscopy of Zebrafish Embryos

The growth and proliferation of zebrafish tumor cells were observed by inverted fluorescence microscopy (IX71, Olympus, Japan) on the day of cell injection and every day after administration, and the presence of new blood vessels was also observed. If active metastasis occurs, fluorescent-labeled tumor cells will appear outside the yolk sac area (head, trunk, tail, etc.).

### 2.9. Statistical Analyses

All the experiments were repeated at least twice. GraphPad Prism 5.0, a commonly used graphics software, was used for data analysis. The histogram selected one-way ANOVA and Dunnett multiple comparison tests and took the average SEM. After cell injection, 10 embryos were taken as a group to prepare single cell suspension, and the number of CM-DI labeled cells was used as the baseline value before drug treatment. The cell proliferation of different concentration groups observed by 3dpt was counted and compared with the baseline value. P < 0.005 (*∗∗∗*) had statistical significance, P < 0.01 (*∗∗*) showed significant difference, and P < 0.05 (*∗*) indicated significant difference.

## 3. Results

### 3.1. In Vitro Tyrosine Kinase Inhibitors Inhibit the Viability of Human Lung Cancer Cell Lines

**Figure 1:**
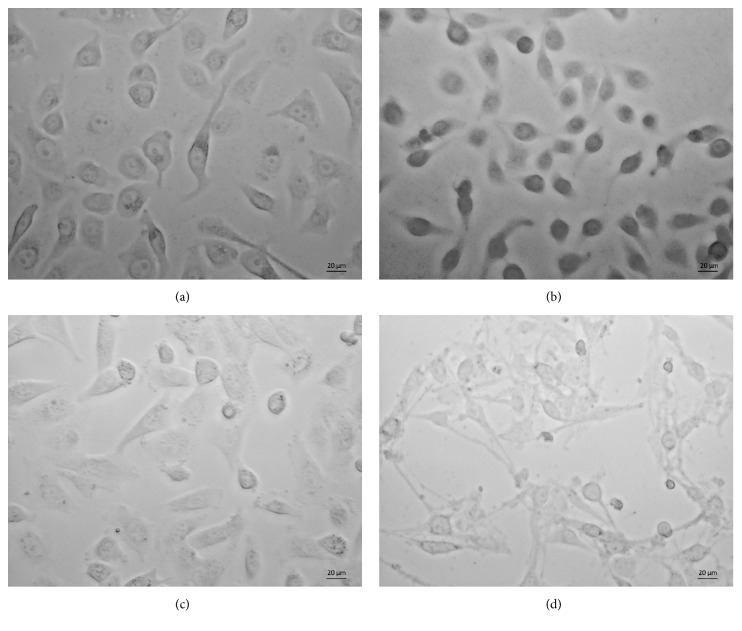
*Establishment of drug-resistant cell lines.* Typical photographs were taken to observe the cell morphology after 24-hour adherence to the 6-well plate. (a) PC9, (b) PC9-Gefitinib-resistant cell line, (c) H1975, (d) H1975-Osimertinib-resistant cell line.

PC9-Gefitinib-resistant cell lines (PC9-GR) and H1975-Osimertinib-resistant cell lines (H1975-OR) were cultured through continuous exposure to Gefitinib and Osimertinib for about six months (Figure 1).

**Figure 2:**
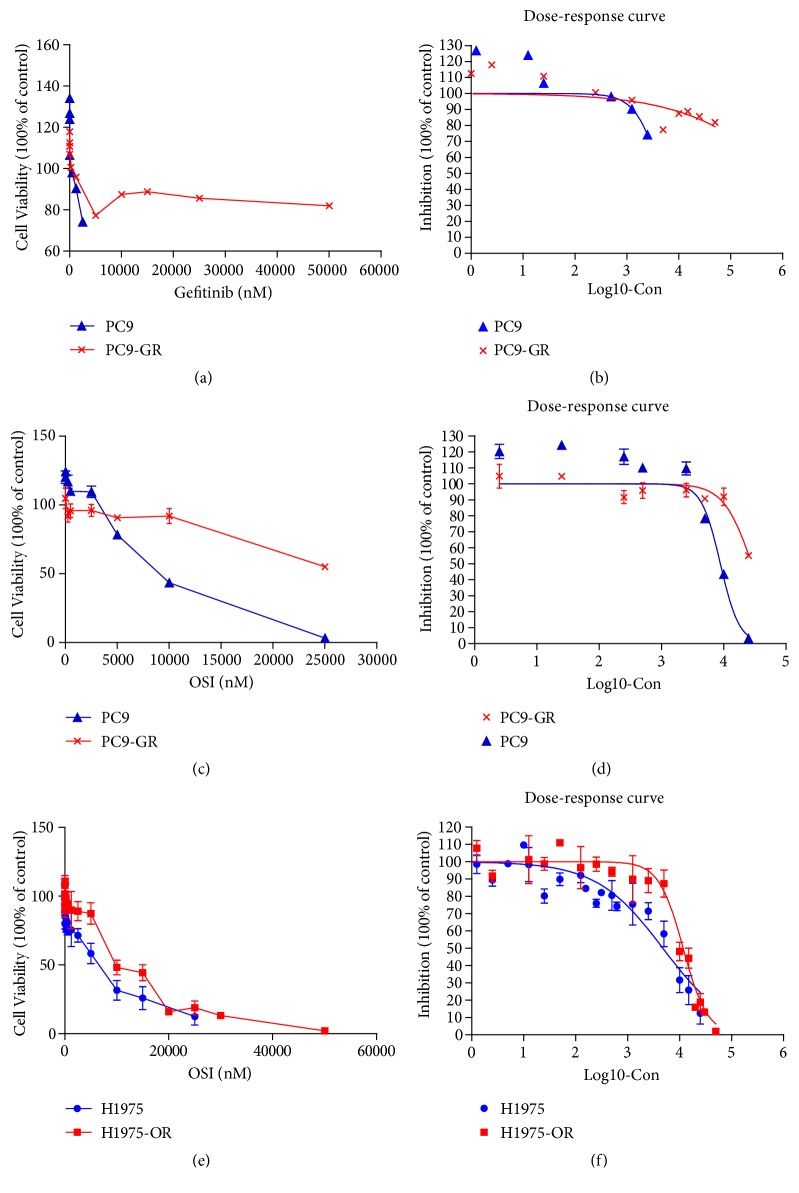
*In vitro cell viability and Osimertinib IC50 assay.* (a) Cell viability of PC9 and PC9-GR cell lines treated with Gefitinib. (b) PC9 and PC9-GR were incubated with Gefitinib at different concentrations for 72 hours. MTT was used to detect the inhibitory effect of Gefitinib on cell proliferation. (c) The cell viability of PC9 and PC9-GR cell lines treated with Osimertinib. (d) PC9 and PC9-GR were incubated with different concentrations of Osimertinib for 72 hours. MTT was used to detect the inhibitory effect of Osimertinib on cell proliferation. (e) The cell viability of H1975 and H1975-OR cell lines treated with Osimertinib. (f) H1975 and H1975-OR were incubated with different concentrations of Osimertinib for 72 hours. MTT was used to detect the inhibitory effect of Osimertinib on cell proliferation.

The cell viability of PC9 and PC9-GR cell lines treated with Gefitinib was measured by MTT assay. It was found that the survival rate of PC9-GR cells did not decrease as significantly as that of PC9 cells after 24 hours of Gefitinib exposure (Figure 2(a)). The IC50 values of Gefitinib for PC9-GR and PC9 cells were 549195 nM and 4568 nM, respectively (Figure 2(b)), with resistance index (RI) exceeding 120. However, with the third-generation EGFR-TKI Osimertinib treatment, the cell viability of both strains decreased (Figure 2(c)). The IC50 values of Osimertinib in PC9-GR and PC9 cells were 28322 nM and 8853 nM, respectively (Figure 2(d)).

H1975 is a common human lung adenocarcinoma cell line. It carries EGFR-L858R sensitive mutation or EGFR-T790M resistant mutation. It is resistant to the first generation of EGFR-TKI Gefitinib but sensitive to the third generation of EGFR-TKI Osimertinib. MTT assays were used to determine the cell viability of H1975 and H1975-OR cell lines treated with Osimertinib (Figure 2(e)). The average IC50 value of Osimertinib for H1975 was 4776 nM through a lot of repeated tests, and that of Osimertinib for H1975-OR was 11740 nM. The difference can be seen from the curve of Figure 2(f).

### 3.2. Antiangiogenic Activity and Antitumor Effect in a Zebrafish Xenograft Model

**Figure 3:**
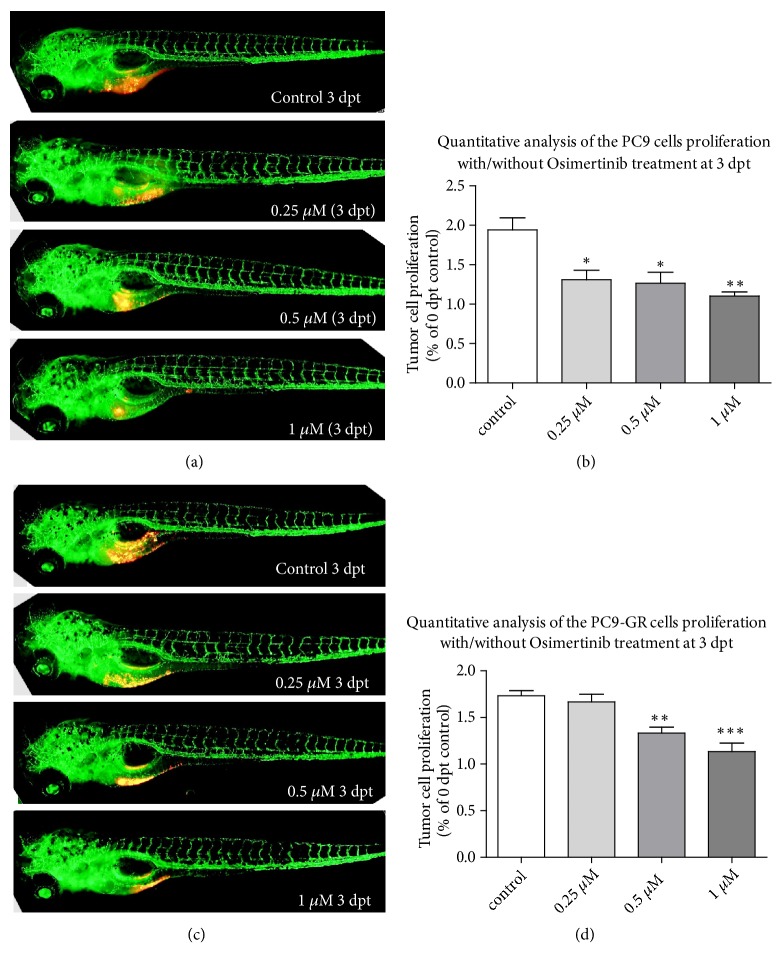
*Lung cancer cells survive in zebrafish larvae and induce angiogenesis.* Zebrafish are labeled as fli-eGFP; the tumor cells were labeled with red fluorescence. (a) PC9 xenograft zebrafish treated with Osimertinib at 24 hpf and observed at 3 dpt. (b) Quantitative analysis of the PC9 cells proliferation with/without Osimertinib treatment at 3 dpt. (c) PC9-GR xenograft zebrafish treated with Osimertinib at 24 hpf and observed at 3 dpt. (d) Quantitative analysis of the PC9-GR cells proliferation with/without Osimertinib treatment at 3 dpt. Hpf: hours postfertilization; dpt: days posttreatment.

Different concentrations of drug treatment have certain effects on the area of subintestinal vessels in zebrafish. This area represents the antiangiogenesis effect of drugs on tumors. The fluorescence intensity after different concentration of drug treatment represents the inhibition rate of tumor growth. In order to evaluate the inhibitory effect of Osimertinib on tumor and angiogenesis in xenograft tumors, the density of PC9 cells was adjusted to 6 × 10^7^cells/ml, and the xenograft model of zebrafish was established by microinjection. Four days after injection, PC9 xenografts proliferated about 1.9 times in zebrafish. In some zebrafish, PC9 cells can induce angiogenesis (Figure 3(a)). Compared with the control group, 0.25*μ*M and 0.5*μ*M Osimertinib could significantly inhibit the proliferation of PC9 cells in vivo (p < 0.05). With the increase of drug concentration, the size of PC9 xenotransplantation tumors was significantly inhibited at 1*μ*M (p < 0.01) (Figure 3(b)).

### 3.3. Osimertinib Inhibits Zebrafish Xenotransplantation of Gefitinib-Resistant Cell Lines

Microinjection of Gefitinib-resistant strain PC9-GR into zebrafish was still used to establish the model. Four days after injection, PC9-GR xenografts proliferated about 1.7 times in zebrafish. PC9-GR cells can induce angiogenesis in some zebrafish (Figure 3(c)). However, unlike the in vivo results of wild-type PC9 cells, only 0.5 and 1*μ*M Osimertinib could inhibit the proliferation of PC9-GR cells in a dose-dependent manner at the same three doses (p < 0.05) (Figure 3(d)). After the first generation of TKI resistance, the Gefitinib-resistant cell line PC9-GR was still sensitive to Osimertinib, but the Gefitinib-sensitive cell line PC9 was more sensitive to Osimertinib (Figures 3(b) and 3(d)). This is consistent with the current EGFR-TKI clinical guidelines recommending the use of third generation of drugs after progress in the use of the first and second generations of drugs.

### 3.4. Osimertinib Inhibits the Growth of Zebrafish Xenografts Produced by H1975 Cells

**Figure 4:**
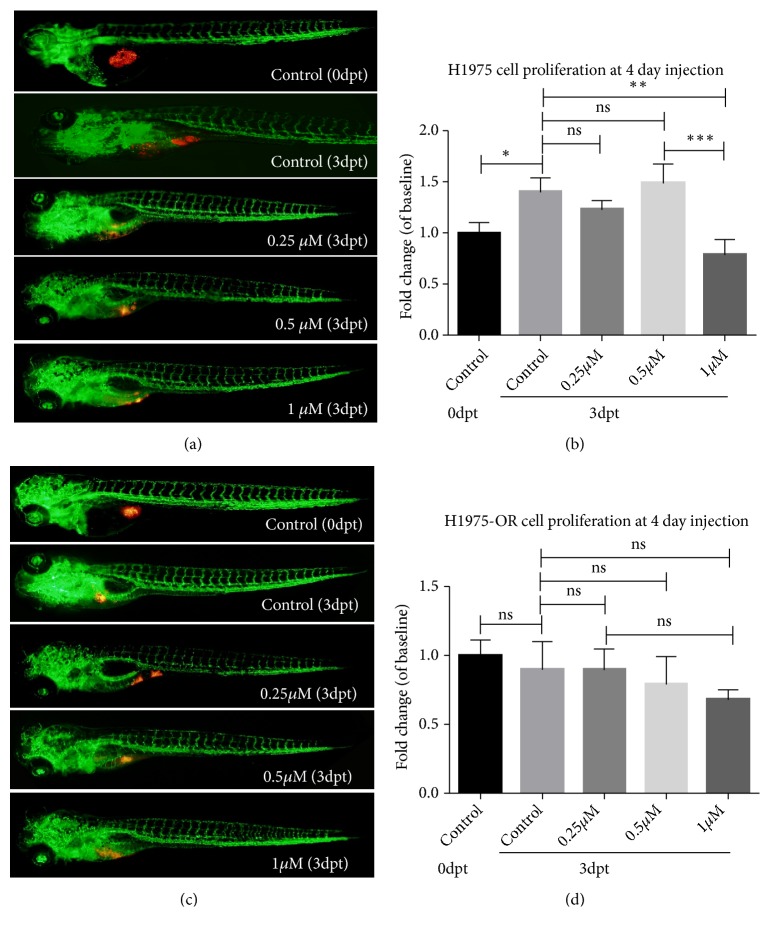
*Osimertinib inhibits the growth of zebrafish xenografts produced by lung cancer cells.* Zebrafish are labeled as fli-eGFP; the tumor cells were labeled with red fluorescence. (a) H1975 xenograft zebrafish treated with Osimertinib at 24 hpf and observed at 3 dpt. (b) Quantitative analysis of the H1975 cells proliferation with/without Osimertinib treatment at 3 dpt. (c) H1975-OR xenograft zebrafish treated with Osimertinib at 24 hpf and observed at 3 dpt. (d) Quantitative analysis of the H1975-OR cells proliferation with/without Osimertinib treatment at 3 dpt. Hpf: hours postfertilization; dpt: days posttreatment.

With the prolongation of injection time, the new vascular foci in the neoplasia area of zebrafish increased (Figure 4(a)). Similarly, the concentration of Osimertinib in H1975 cells carrying EGFR L858R/T790M mutant was measured in vivo. The initial amount of H1975 cells injected was still 6 × 10^7^cells/ml, but after 4 days of injection, the number of H1975 cells exceeded 1.4 times the initial amount, showing a significant difference (Figure 4(b)). With different concentration gradients, neither 0.25*μ*M nor 0.5*μ*M could inhibit the proliferation of H1975 cells in zebrafish, or even the proliferation of tumor cells under 0.5*μ*M Osimertinib, which was similar to the control group. Only when the concentration of Osimertinib reached 1*μ*M, the proliferation of H1975 in zebrafish was significantly inhibited, and the P value was less than 0.01(Figure 4(b)).

### 3.5. Drug Resistance in Xenograft Tumors of Osimertinib

Three generations of EGFR-TKI Osimertinib H1975-OR resistant strains were established after six months of continuous exposure to about 2*μ*M concentration of Osimertinib. Comparing the drug resistance of the third generation of drug-resistant strains in vivo (Figure 4(c)), no significant inhibition of the growth of H1975-OR cells was observed in the three concentration groups of 0.25*μ*M, 0.5*μ*M, and 1*μ*M. The growth of tumor cells in the cancer area was very close to that in the control group, indicating that the drug-resistant H1975-OR cell lines were not sensitive to Osimertinib (Figure 4(d)).

## 4. Discussion

About two-thirds of NSCLC patients carry EGFR mutations, which can benefit significantly from tyrosinase inhibitor TKI [22] because targeted drugs directly bind to the intracellular domain of EGFR to inhibit downstream signaling pathways. However, more than half of the EGFR-positive patients developed drug resistance after treatment with Gefitinib, Erlotinib, and Afatinib for about 12 months. Repeated tumor biopsy comprehensively analyzed the drug resistance mechanism of advanced patients with EGFR-TKIs. About 60%-70% of the cases have identified three main types of drug resistance mechanisms [23]: (1) the insurgence of secondary mutations in the EGFR gene, most common among which is T790M, and C797S mutation, which was first discovered after the use of three generations of TKI; (2) phenotypic transformation, such as AXL upregulation in the context of EMT; (3) activation of alternative pathways, including MET and ERBB2 amplification.

Osimertinib, as the only third-generation EGFR-TKI approved by the FDA in the United States, is effectively used in patients with EGFR mutation and T790M mutation [8, 9]. It irreversibly inhibited tyrosine kinase activity and had no adverse effect on wild-type EGFR [24]. However, 40% of patients treated with Osimertinib produce C797S mutations, and there are other different mechanisms of drug resistance that lead to the recurrence of disease progression [11]. In this study, we start with establishing drug-resistant cell lines of Gefitinib and Osimertinib to evaluate cell proliferation in vitro and the inhibition of different drug concentration gradients on cancer cells, so as to verify the resistance of Gefitinib-resistant strains and Osimertinib-resistant strains. From the dose-response curve, we can see that the curve of drug-resistant cell lines is on the right side, which is obviously different from that of sensitive cell lines.

Previous studies on PDX mainly focused on the comparison of biomarkers of tumors. In this study, zebrafish xenotransplantation models were used as research carriers to establish models by transplanting different tumor cell lines into zebrafish. The proliferation ability of drug-resistant and sensitive strains was evaluated by observing the proliferation of tumor cells in vivo visually and clearly by fluorescence staining. Although there were no antiangiogenesis drugs in this experiment, we could evaluate the inhibitory effect of different concentrations of Osimertinib on angiogenesis by comparing the size of neovascularization in zebrafish cancer foci caused by transplantation of different tumor cell lines.

We first focused on comparing the tumorigenicity of PC9 and PC9-GR cell lines after xenotransplantation into zebrafish. Four days after injecting cells, sensitive strains and first-generation TKI Gefitinib-resistant strains proliferated 1.9 and 1.7 times in zebrafish, respectively, which fully verified the proliferation ability of these two human lung cancer cells in vivo. One of the most basic characteristics of cancer cells is sustaining proliferative signaling [25], which requires the activation of downstream growth-related signaling pathways, including the well-known RAS pathway. The growth rate of Gefitinib-resistant strains in vivo was 1.7 times faster than that of the control group, but slower than that of sensitive strains, probably due to the influence of EGFR pathway after drug resistance. Angiogenesis was observed in both resistant and sensitive strains of Gefitinib zebrafish xenografts, which is also a major feature of malignant tumors and promotes angiogenesis.

Previous studies have demonstrated that Osimertinib monotherapy could be effective for patients with EGFR mutations, especially in patients with T790M mutations, and started to inhibit wild-type EGFR more significantly in patients at high dose levels [8]. In this study, only three concentration gradients, 0.25*μ*M, 0.5*μ*M, and 1*μ*M, were set up in the small zebrafish xenotransplantation model. Different doses of Osimertinib could significantly inhibit the proliferation of wild-type PC9 cells in vivo. However, in PC9-GR group, 0.25*μ*M Osimertinib did not seem to have any effect on the proliferation of Gefitinib-resistant PC9-GR strain, and it could inhibit the growth of tumors only when the drug concentration increased to 0.5*μ*M. This is consistent with the results of clinical trials [8, 26]. The Gefitinib-resistant cell line PC9-GR is sensitive to Osimertinib, but in this experiment, the Gefitinib-sensitive cell line PC9 is more sensitive to Osimertinib in zebrafish.

In recent years, a number of large clinical studies [8, 10, 26] have proved that Osimertinib had significantly greater efficacy and safety than platinum-containing chemotherapy in patients whose disease had progressed during first-line EGFR-TKI therapy.  Figure 3 simulates clinical drug use by comparing the tumor inhibition of drug-resistant strain H1975-OR and susceptible strain H1975 in zebrafish. Four days after injection of H1975 cells into zebrafish, the proliferation of H1975 cells was 1.4 times, which confirmed that the tumor cells carrying the common TKI-resistant mutation T790M still maintained the proliferation ability, but the third generation of TKI Osimertinib-resistant strain H1975-OR did not significantly proliferate compared with the control group. The weak proliferation of drug-resistant and contrast-sensitive strains in vivo may be related to the changes of EGFR-related pathways mentioned above. Although C797S mutation, RAS mutation, and MET amplification are all considered to be the causes of Osimertinib resistance, loss of EGFR expression [27] has been identified as a potential resistant mechanism of Osimertinib in H1975-OR cells, suggesting that patients may no longer benefit from EGFR-TKIs or EGFR antibodies [28].

After treatment with three concentrations of Osimertinib, a significant difference in the proliferation of H1975 in zebrafish and the control group was observed only at the concentration of 1*μ*M, with tumor suppression in vivo. After xenotransplantation of Osimertinib-resistant strain H1975-OR into zebrafish, three concentrations of Osimertinib did not significantly inhibit the growth of zebrafish tumors. The comparison showed that the proliferation of H1975-OR cells decreased gradually with the increase of Osimertinib concentration, but there was no statistical significance. This may be because the concentration set in the experiment is too low to inhibit the proliferation of tumor cells in zebrafish. Osimertinib-resistant cells were not sensitive to Osimertinib in vivo. The successful establishment of xenotransplantation model of drug-resistant cell lines will help to find effective therapeutic drugs for third-generation TKI-resistant patients in zebrafish, a model similar to the human body, which will also be our future work plan.

## 5. Conclusions

In summary, we have established drug-resistant strains of Gefitinib and Osimertinib by continuously adding drugs. Zebrafish were selected as xenotransplantation models to compare the effects of different concentrations of Osimertinib on proliferation and angiogenesis of zebrafish after transplantation of different lung cancer cell lines. It was confirmed that Osimertinib could inhibit the proliferation of tumor cells with EGFR mutation and T790M resistance mutation in zebrafish, which was consistent with the clinical research conclusion. The successful establishment of this third-generation EGFR-TKI drug-resistant xenograft model can be a rapid tool for further understanding the mechanism of Osimertinib (AZD9291) resistance and exploring the follow-up drug therapy for third-generation EGFR-TKI resistant patients. It also promotes the use of zebrafish xenotransplantation model as a real-time drug screening platform for clinical lung cancer patients. And it provides individualized therapy that suits the circumstances of each patient on the basis of guidelines.

## Data Availability

The data used to support the findings of this study are included within the article.

## Abbreviations

NSCLC: Non-small cell lung cancer
TKI: Tyrosine kinase inhibitor
EGFR: Epidermal growth factor receptor
ALK: Anaplastic lymphoma kinase
ROS1: ROS1 protooncogene receptor tyrosine kinase
BRAF: Serine/threonine protein kinase b-Raf
T790M: Thr790Met
PDX: Patient-derived xenotransplantation
zPDX: Zebrafish xenotransplantation model
DMSO: Dimethyl sulfoxide
FBS: Fetal bovine serum
Hpf: Hours postfertilization
dpt: Days posttreatment.

## References

[1] Bray F., Ferlay J., Soerjomataram I. (2018). Global cancer statistics 2018: GLOBOCAN estimates of incidence and mortality worldwide for 36 cancers in 185 countries. *CA: A Cancer Journal for Clinicians*.

[2] Kris M. G., Johnson B. E., Berry L. D. (2014). Using multiplexed assays of oncogenic drivers in lung cancers to select targeted drugs. *The Journal of the American Medical Association*.

[3] Barr N. K., Zanwijk N. V., Soo R. A. (2015). Molecular targeted therapy in the treatment of advanced stage non-small cell lung cancer (NSCLC). *Respirology*.

[4] Zhang Z., Stiegler A. L., Boggon T. J., Kobayashi S., Halmos B. (2010). EGFR-mutated lung cancer: a paradigm of molecular oncology. *Oncotarget*.

[5] Maemondo M., Inoue A., Kobayashi K. (2010). Gefitinib or chemotherapy for non-small-cell lung cancer with mutated EGFR. *The New England Journal of Medicine*.

[6] Pao W., Miller V. A., Politi K. A. (2005). Acquired resistance of lung adenocarcinomas to gefitinib or erlotinib is associated with a second mutation in the EGFR kinase domain. *PLoS Medicine*.

[7] Cross D. A. E., Ashton S. E., Ghiorghiu S. (2014). AZD9291, an irreversible EGFR TKI, overcomes T790M-mediated resistance to EGFR inhibitors in lung cancer. *Cancer Discovery*.

[8] Jänne P. A., Chih-Hsin Yang J., Kim D.-W. (2015). AZD9291 in EGFR inhibitor-resistant non-small-cell lung cancer. *The New England Journal of Medicine*.

[9] Sullivan I., Planchard D. (2016). Osimertinib in the treatment of patients with epidermal growth factor receptor T790M mutation-positive metastatic non-small cell lung cancer: Clinical trial evidence and experience. *Therapeutic Advances in Respiratory Disease*.

[10] Mok T. S., Wu Y., Ahn M. (2017). Osimertinib or platinum–pemetrexed in EGFR T790M–positive lung cancer. *The New England Journal of Medicine*.

[11] Thress K. S., Paweletz C. P., Felip E. (2015). Acquired EGFR C797S mutation mediates resistance to AZD9291 in non-small cell lung cancer harboring EGFR T790M. *Nature Medicine*.

[12] Lim S. M., Syn N. L., Cho B. C., Soo R. A. (2018). Acquired resistance to EGFR targeted therapy in non-small cell lung cancer: Mechanisms and therapeutic strategies. *Cancer Treatment Reviews*.

[13] DeBord L. C., Pathak R. R., Villaneuva M., Liu H. C., Harrington D. A. (2018). The chick chorioallantoic membrane (CAM) as a versatile patient-derived xenograft (PDX) platform for precision medicine and preclinical research. *American Journal of Cancer Research*.

[14] Stewart E. L., Mascaux C., Pham N.-A. (2015). Clinical utility of patient-derived xenografts to determine biomarkers of prognosis and map resistance pathways in EGFR-mutant lung adenocarcinoma. *Journal of Clinical Oncology*.

[15] Yamazaki S., Vicini P., Shen Z. (2012). Pharmacokinetic/pharmacodynamic modeling of crizotinib for anaplastic lymphoma kinase inhibition and antitumor efficacy in human tumor xenograft mouse models. *The Journal of Pharmacology and Experimental Therapeutics*.

[16] Schier A. F. (2013). Genomics: Zebrafish earns its stripes. *Nature*.

[17] Raldúa D., Piña B. (2014). In vivo zebrafish assays for analyzing drug toxicity. *Expert Opinion on Drug Metabolism & Toxicology*.

[18] Veinotte C. J., Dellaire G., Berman J. N. (2014). Hooking the big one: the potential of zebrafish xenotransplantation to reform cancer drug screening in the genomic era. *Disease Models & Mechanisms*.

[19] Bentley V. L., Veinotte C. J., Corkery D. P. (2015). Focused chemical genomics using zebrafish xenotransplantation as a pre-clinical therapeutic platform for T-cell acute lymphoblastic leukemia. *Haematologica*.

[20] Lin J., Zhang W., Zhao J.-J. (2016). A clinically relevant in vivo zebrafish model of human multiple myeloma to study preclinical therapeutic efficacy. *Blood*.

[21] Fior R., Póvoa V., Mendes R. V. (2017). Single-cell functional and chemosensitive profiling of combinatorial colorectal therapy in zebrafish xenografts. *Proceedings of the National Acadamy of Sciences of the United States of America*.

[22] Mitsudomi T., Kosaka T., Yatabe Y. (2006). Biological and clinical implications of EGFR mutations in lung cancer. *International Journal of Clinical Oncology*.

[23] Morgillo F., Della Corte C. M., Fasano M., Ciardiello F. (2016). Mechanisms of resistance to EGFR-targeted drugs: Lung cancer. *ESMO Open*.

[24] Yap T. A., Popat S. (2014). Toward precision medicine with next-generation EGFR inhibitors in non-small-cell lung cancer. *Pharmacogenomics and Personalized Medicine*.

[25] Hanahan D., Weinberg R. A. (2011). Hallmarks of cancer: the next generation. *Cell*.

[26] Goss G., Tsai C.-M., Shepherd F. A. (2016). Osimertinib for pretreated EGFR Thr790Met-positive advanced non-small-cell lung cancer (AURA2): a multicentre, open-label, single-arm, phase 2 study. *The Lancet Oncology*.

[27] Tang Z.-H., Jiang X.-M., Guo X., Fong C. M. V., Chen X., Lu J.-J. (2016). Characterization of osimertinib (AZD9291)-resistant non-small cell lung cancer NCI-H1975/OSIR cell line. *Oncotarget *.

[28] Sequist L. V., Waltman B. A., Dias-Santagata D. (2011). Genotypic and histological evolution of lung cancers acquiring resistance to EGFR inhibitors. *Science Translational Medicine*.

